# Bis[2,4-dibromo-6-(ethyl­imino­methyl)phenolato-κ^2^ *N*,*O*]cobalt(II)

**DOI:** 10.1107/S1600536810032174

**Published:** 2010-08-18

**Authors:** Chunyan Li, Rui Li, Shufang Zhang

**Affiliations:** a College of Health Science, Wuhan Institute of Physical Education, Wuhan 430079, People’s Republic of China

## Abstract

In the title compound, [Co(C_9_H_8_Br_2_NO)_2_], the Co^II^ atom, located on a twofold axis, is in a pseudo-tetra­hedral environment, with two bidentate 2,4-dibromo-6-(ethyl­imino­meth­yl)phenolate Schiff base ligands acting as chelates through their phenolate O and azomethine N atoms. C—H⋯O hydrogen bonds link the complex mol­ecules to form a chain parallel to the *b* axis.

## Related literature

For related Lewis base adducts, see: Akitsu *et al.* (2005 ▶); Bahron *et al.* (1994 ▶); Bermejo *et al.* (1996 ▶); Elerman *et al.* (1996 ▶); Groombridge *et al.* (1992 ▶); Li *et al.* (2008 ▶); Maneiro *et al.* (2001 ▶); Qiu *et al.* (2007 ▶). For a related structure, see: Jiang *et al.* (2008 ▶). For standard bond-distance values, see: Allen *et al.* (1987 ▶).
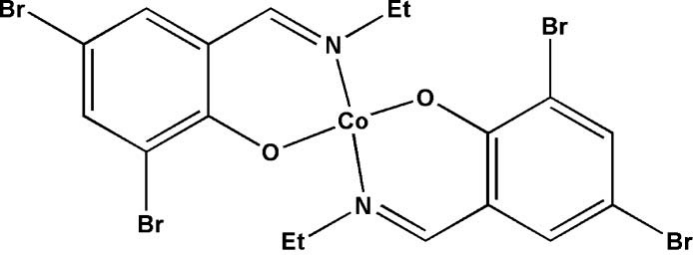


## Experimental

### 

#### Crystal data

[Co(C_9_H_8_Br_2_NO)_2_]
*M* *_r_* = 670.90
Monoclinic, 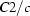

*a* = 22.116 (3) Å
*b* = 4.8645 (5) Å
*c* = 19.652 (2) Å
β = 100.038 (3)°
*V* = 2081.9 (4) Å^3^
*Z* = 4
Mo *K*α radiation
μ = 8.52 mm^−1^
*T* = 298 K
0.30 × 0.21 × 0.11 mm

#### Data collection

Bruker SMART CCD area-detector diffractometer
Absorption correction: multi-scan (*SADABS*; Bruker, 2000 ▶) *T*_min_ = 0.089, *T*_max_ = 0.392
6537 measured reflections
2028 independent reflections
1607 reflections with *I* > 2σ(*I*)
*R*_int_ = 0.099

#### Refinement

*R*[*F* ^2^ > 2σ(*F* ^2^)] = 0.060
*wR*(*F* ^2^) = 0.161
*S* = 1.00
2028 reflections
124 parameters
H-atom parameters constrained
Δρ_max_ = 1.39 e Å^−3^
Δρ_min_ = −1.08 e Å^−3^

### 

Data collection: *SMART* (Bruker, 2000 ▶); cell refinement: *SAINT* (Bruker, 2000 ▶); data reduction: *SAINT*; program(s) used to solve structure: *SHELXS97* (Sheldrick, 2008 ▶); program(s) used to refine structure: *SHELXL97* (Sheldrick, 2008 ▶); molecular graphics: *ORTEPIII* (Burnett & Johnson, 1996 ▶), *ORTEP-3 for Windows* (Farrugia, 1997 ▶) and *PLATON* (Spek, 2009 ▶).; software used to prepare material for publication: *SHELXL97*.

## Supplementary Material

Crystal structure: contains datablocks global, I. DOI: 10.1107/S1600536810032174/dn2591sup1.cif

Structure factors: contains datablocks I. DOI: 10.1107/S1600536810032174/dn2591Isup2.hkl

Additional supplementary materials:  crystallographic information; 3D view; checkCIF report

## Figures and Tables

**Table 1:** Hydrogen-bond geometry (Å, °)

*D*—H⋯*A*	*D*—H	H⋯*A*	*D*⋯*A*	*D*—H⋯*A*
C8—H8*A*⋯O1^i^	0.97	2.49	3.389 (8)	154

Symmetry code: (i) 
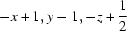
.

**Fig. 1.**
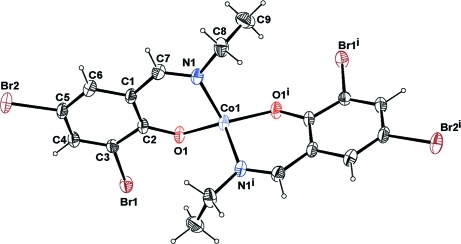
The structure of the title compound (I), with the atom labeling scheme. Ellipsoids are drawn at the 30% probability level. H atoms are represented as small spheres of arbitrary radii. [Symmetry code: (i) 1 - x, y, 1/2 - z.]

**Fig. 2.**
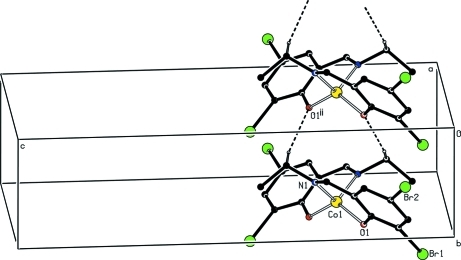
Partial packing view showing the chain formed through C—H···O hydrogen bonds. H atoms not involved in hydrogen bondings have been omitted for clarity. H bonds are shown as dashed lines. [Symmetry code: (ii) -x + 1, y - 1, -z + 1/2.]

## supplementary crystallographic information

### Comment

The Lewis base adducts of the 3,5-dibromosalicylidene group that are derived from the condensation of 3,5-dibromosalicylaldehyde and various primary amine are very interesting in a large number of transition metal complexes (Qiu *et al.*, 2007; Akitsu *et al.*, 2005; Maneiro *et al.*, 2001; Bermejo *et al.*, 1996). Recently, some mononuclear cobalt(II) compounds of Schiff base ligands derived from the condensation of salicylaldehyde with ethyl-, propyl- and butylamine have been structurally characterized (Li *et al.*, 2008; Elerman *et al.*, 1996; Bahron *et al.*, 1994; Groombridge *et al.*, 1992). As an extension of this work, the crystal structure of the title compound, (I), is reported here.

In (I), the Co atoms, located on a two fold axis, have pseudo-tetrahedral coordination environments with two bidentate Schiff base ligands,derived from the condensation of 3,5-dibromosalicylaldehyde and ethylamine, acting as chelates through their phenolate O and azomethine N atoms (Fig. 1). The structure is closely related to the Bis{2-[(*E*)-benzyliminomethyl]-4,6-dibromophenolato-κ^2^*N*, *O*}cobalt(II) compound (Jiang *et al.*, 2008). The C7═N1 bond length of 1.287 (7) Å is similar to that of 1.288 (7) Å observed in the previously reported compound of Schiff base ligand, which was derived from the condensation of salicylaldehyde and isopropylamine (Elerman *et al.*, 1996). The angle between the two O1—Co1—N1 planes of the molecule is equal to 82.80°. All bond lengths are within normal ranges (Allen *et al.*, 1987).

In the crystal structure, the molecules are linked *via* intermolecular C—H···O hydrogen bonds forming a chain parallel to the b axis (Table 1, Fig. 2).

### Experimental

3,5-Dibromosalicyladehyde (560 mg, 2 mmol) and ethylamine (90 mg, 2 mmol) were dissolved in methanol (25 ml). The mixture was stirred for 30 min to give an orange solution, which was added to a methanol solution (15 ml) of Co(NO_3_)_2_.6H_2_O (280 mg, 1 mmol). The mixture was stirred for another 20 min at room temperature to give a red solution and then filtered. The filtrate was kept in air for 5 d, forming red blocky crystals. The crystals were isolated, washed three times with distilled water and dried in a vacuum desiccator containing anhydrous CaCl_2_ (yield 66%). Analysis calculated for C_18_H_16_Br_4_CoN_2_O_2_: C 32.23, H 2.40, N 4.18%; found: C 32.11, H 2.55, N 4.00%. IR (KBr, cm^-1^): 3447, 2956, 2925, 2868, 2363, 1736, 1616, 1581, 1505, 1436, 1406, 1343, 1309, 1210, 1156, 1090, 1058, 974, 863, 840, 750, 708, 606, 534, 477.

### Refinement

All the H atoms were placed in geometrically idealized positions and constrained to ride on their parent atoms, with C—H distances of 0.93–0.97 Å, and with *U*_iso_(H) = 1.2*U*_eq_(carrier) or 1.5*U*_eq_(methyl groups).

### Crystal data

**Table:**

[Co(C_9_H_8_Br_2_NO)_2_]	*F*(000) = 1284
*M**_r_* = 670.90	*D*_x_ = 2.140 Mg m^−^^3^
Monoclinic, *C*2/*c*	Mo *K*α radiation, λ = 0.71073 Å
Hall symbol: -C 2yc	Cell parameters from 2472 reflections
*a* = 22.116 (3) Å	θ = 2.6–27.2°
*b* = 4.8645 (5) Å	µ = 8.52 mm^−^^1^
*c* = 19.652 (2) Å	*T* = 298 K
β = 100.038 (3)°	Block, red
*V* = 2081.9 (4) Å^3^	0.30 × 0.21 × 0.11 mm
*Z* = 4	

### Data collection

**Table:**

Bruker SMART CCD area-detector diffractometer	2028 independent reflections
Radiation source: fine-focus sealed tube	1607 reflections with *I* > 2σ(*I*)
graphite	*R*_int_ = 0.099
φ and ω scans	θ_max_ = 26.0°, θ_min_ = 1.9°
Absorption correction: multi-scan (*SADABS*; Bruker, 2000)	*h* = −26→26
*T*_min_ = 0.089, *T*_max_ = 0.392	*k* = −6→5
6537 measured reflections	*l* = −24→22

### Refinement

**Table:**

Refinement on *F*^2^	Primary atom site location: structure-invariant direct methods
Least-squares matrix: full	Secondary atom site location: difference Fourier map
*R*[*F*^2^ > 2σ(*F*^2^)] = 0.060	Hydrogen site location: inferred from neighbouring sites
*wR*(*F*^2^) = 0.161	H-atom parameters constrained
*S* = 1.00	*w* = 1/[σ^2^(*F*_o_^2^) + (0.0865*P*)^2^] where *P* = (*F*_o_^2^ + 2*F*_c_^2^)/3
2028 reflections	(Δ/σ)_max_ < 0.001
124 parameters	Δρ_max_ = 1.39 e Å^−^^3^
0 restraints	Δρ_min_ = −1.08 e Å^−^^3^

### Special details

**Table:**

Geometry. All e.s.d.'s (except the e.s.d. in the dihedral angle between two l.s. planes) are estimated using the full covariance matrix. The cell e.s.d.'s are taken into account individually in the estimation of e.s.d.'s in distances, angles and torsion angles; correlations between e.s.d.'s in cell parameters are only used when they are defined by crystal symmetry. An approximate (isotropic) treatment of cell e.s.d.'s is used for estimating e.s.d.'s involving l.s. planes.
Refinement. Refinement of *F*^2^ against ALL reflections. The weighted *R*-factor *wR* and goodness of fit *S* are based on *F*^2^, conventional *R*-factors *R* are based on *F*, with *F* set to zero for negative *F*^2^. The threshold expression of *F*^2^ > σ(*F*^2^) is used only for calculating *R*-factors(gt) *etc*. and is not relevant to the choice of reflections for refinement. *R*-factors based on *F*^2^ are statistically about twice as large as those based on *F*, and *R*- factors based on ALL data will be even larger.

### Fractional atomic coordinates and isotropic or equivalent isotropic displacement parameters (Å^2^)

**Table:**

	*x*	*y*	*z*	*U*_iso_*/*U*_eq_	
Co1	0.5000	0.9495 (3)	0.2500	0.0396 (3)	
N1	0.44643 (19)	0.7331 (11)	0.3012 (2)	0.0394 (11)	
O1	0.43303 (17)	1.1213 (9)	0.1898 (2)	0.0423 (10)	
Br1	0.36561 (3)	1.36837 (14)	0.05745 (3)	0.0477 (3)	
Br2	0.18010 (3)	0.63810 (17)	0.10502 (4)	0.0595 (3)	
C1	0.3544 (2)	0.8138 (12)	0.2155 (3)	0.0354 (12)	
C2	0.3778 (2)	1.0161 (13)	0.1750 (3)	0.0343 (12)	
C3	0.3371 (3)	1.1041 (12)	0.1146 (3)	0.0354 (13)	
C4	0.2796 (2)	0.9972 (13)	0.0949 (3)	0.0398 (13)	
H4	0.2549	1.0568	0.0544	0.048*	
C5	0.2587 (2)	0.8016 (14)	0.1352 (3)	0.0405 (14)	
C6	0.2942 (3)	0.7099 (14)	0.1949 (3)	0.0438 (14)	
H6	0.2789	0.5792	0.2219	0.053*	
C7	0.3891 (3)	0.6970 (13)	0.2770 (3)	0.0406 (13)	
H7	0.3677	0.5820	0.3023	0.049*	
C8	0.4728 (3)	0.5890 (14)	0.3657 (3)	0.0508 (16)	
H8A	0.5098	0.4929	0.3593	0.061*	
H8B	0.4437	0.4538	0.3767	0.061*	
C9	0.4878 (4)	0.7869 (17)	0.4243 (3)	0.064 (2)	
H9A	0.5131	0.9319	0.4116	0.096*	
H9B	0.5094	0.6928	0.4642	0.096*	
H9C	0.4505	0.8634	0.4349	0.096*	

### Atomic displacement parameters (Å^2^)

**Table:**

	*U*^11^	*U*^22^	*U*^33^	*U*^12^	*U*^13^	*U*^23^
Co1	0.0224 (5)	0.0503 (8)	0.0437 (6)	0.000	−0.0009 (4)	0.000
N1	0.026 (2)	0.043 (3)	0.048 (3)	0.006 (2)	0.0011 (19)	0.001 (2)
O1	0.0245 (19)	0.047 (3)	0.051 (2)	−0.0072 (16)	−0.0047 (17)	0.0032 (19)
Br1	0.0393 (4)	0.0488 (5)	0.0511 (4)	−0.0047 (2)	−0.0036 (3)	0.0089 (3)
Br2	0.0276 (4)	0.0697 (6)	0.0769 (5)	−0.0128 (3)	−0.0030 (3)	−0.0035 (4)
C1	0.022 (2)	0.040 (3)	0.043 (3)	0.002 (2)	0.000 (2)	−0.006 (2)
C2	0.026 (2)	0.032 (3)	0.042 (3)	0.003 (2)	−0.004 (2)	−0.006 (2)
C3	0.029 (3)	0.041 (4)	0.035 (3)	0.002 (2)	0.003 (2)	−0.002 (2)
C4	0.031 (3)	0.043 (4)	0.043 (3)	0.003 (2)	−0.003 (2)	−0.005 (3)
C5	0.022 (2)	0.051 (4)	0.046 (3)	0.000 (2)	−0.001 (2)	−0.008 (3)
C6	0.029 (3)	0.044 (4)	0.059 (4)	−0.003 (3)	0.010 (3)	−0.002 (3)
C7	0.032 (3)	0.041 (4)	0.048 (3)	−0.002 (2)	0.007 (2)	0.003 (3)
C8	0.039 (3)	0.049 (4)	0.058 (4)	0.006 (3)	−0.007 (3)	0.010 (3)
C9	0.071 (5)	0.077 (6)	0.044 (4)	0.016 (4)	0.009 (3)	0.009 (4)

### Geometric parameters (Å, °)

**Table:**

Co1—O1^i^	1.918 (4)	C3—C4	1.366 (8)
Co1—O1	1.918 (4)	C4—C5	1.370 (9)
Co1—N1^i^	1.985 (5)	C4—H4	0.9300
Co1—N1	1.985 (5)	C5—C6	1.367 (9)
N1—C7	1.287 (7)	C6—H6	0.9300
N1—C8	1.477 (8)	C7—H7	0.9300
O1—C2	1.310 (6)	C8—C9	1.493 (10)
Br1—C3	1.886 (6)	C8—H8A	0.9700
Br2—C5	1.909 (5)	C8—H8B	0.9700
C1—C6	1.414 (8)	C9—H9A	0.9600
C1—C2	1.419 (8)	C9—H9B	0.9600
C1—C7	1.432 (8)	C9—H9C	0.9600
C2—C3	1.424 (8)		
			
O1^i^—Co1—O1	128.3 (3)	C6—C5—C4	121.4 (5)
O1^i^—Co1—N1^i^	94.52 (17)	C6—C5—Br2	119.2 (5)
O1—Co1—N1^i^	112.53 (19)	C4—C5—Br2	119.4 (4)
O1^i^—Co1—N1	112.53 (19)	C5—C6—C1	120.0 (6)
O1—Co1—N1	94.52 (17)	C5—C6—H6	120.0
N1^i^—Co1—N1	116.0 (3)	C1—C6—H6	120.0
C7—N1—C8	118.0 (5)	N1—C7—C1	127.3 (6)
C7—N1—Co1	121.5 (4)	N1—C7—H7	116.3
C8—N1—Co1	120.4 (4)	C1—C7—H7	116.3
C2—O1—Co1	123.9 (4)	N1—C8—C9	110.9 (6)
C6—C1—C2	120.3 (5)	N1—C8—H8A	109.5
C6—C1—C7	116.0 (6)	C9—C8—H8A	109.5
C2—C1—C7	123.7 (5)	N1—C8—H8B	109.5
O1—C2—C1	124.3 (5)	C9—C8—H8B	109.5
O1—C2—C3	119.8 (5)	H8A—C8—H8B	108.0
C1—C2—C3	115.9 (5)	C8—C9—H9A	109.5
C4—C3—C2	122.9 (6)	C8—C9—H9B	109.5
C4—C3—Br1	118.8 (4)	H9A—C9—H9B	109.5
C2—C3—Br1	118.3 (4)	C8—C9—H9C	109.5
C3—C4—C5	119.5 (5)	H9A—C9—H9C	109.5
C3—C4—H4	120.2	H9B—C9—H9C	109.5
C5—C4—H4	120.2		

Symmetry codes: (i) −*x*+1, *y*, −*z*+1/2.

### Hydrogen-bond geometry (Å, °)

**Table:**

*D*—H···*A*	*D*—H	H···*A*	*D*···*A*	*D*—H···*A*
C8—H8A···O1^ii^	0.97	2.49	3.389 (8)	154.

Symmetry codes: (ii) −*x*+1, *y*−1, −*z*+1/2.
